# Supplementary material for: Smooth Interpolating Curves with Local Control and Monotone Alternating Curvature
Source: Comput Graph Forum. 2022 Oct 6;41(5):25–38. doi: 10.1111/cgf.14600 (PMC9827861; doi:10.1111/cgf.14600)
Supplement: Supplementary file 1 — Supplement Material [file CGF-41-25-s001.zip › Local-Smooth-Interpolating-MonoCurvature/extern/clothoids/submodules/Utils/docs/api-cpp/program_listing_file_format.cc.html]

Program Listing for File format.cc — Utils v1.2

### Navigation

- index
- toc
- Utils »
- Program Listing for File format.cc

# Program Listing for File format.cc¶

↰ Return to documentation for file (`format.cc`)

```
// Formatting library for C++
//
// Copyright (c) 2012 - 2016, Victor Zverovich
// All rights reserved.
//
// For the license information refer to format.h.

#include "fmt/format-inl.h"

FMT_BEGIN_NAMESPACE
namespace detail {

// DEPRECATED!
template <typename T = void> struct basic_data {
  FMT_API static constexpr const char digits[100][2] = {
      {'0', '0'}, {'0', '1'}, {'0', '2'}, {'0', '3'}, {'0', '4'}, {'0', '5'},
      {'0', '6'}, {'0', '7'}, {'0', '8'}, {'0', '9'}, {'1', '0'}, {'1', '1'},
      {'1', '2'}, {'1', '3'}, {'1', '4'}, {'1', '5'}, {'1', '6'}, {'1', '7'},
      {'1', '8'}, {'1', '9'}, {'2', '0'}, {'2', '1'}, {'2', '2'}, {'2', '3'},
      {'2', '4'}, {'2', '5'}, {'2', '6'}, {'2', '7'}, {'2', '8'}, {'2', '9'},
      {'3', '0'}, {'3', '1'}, {'3', '2'}, {'3', '3'}, {'3', '4'}, {'3', '5'},
      {'3', '6'}, {'3', '7'}, {'3', '8'}, {'3', '9'}, {'4', '0'}, {'4', '1'},
      {'4', '2'}, {'4', '3'}, {'4', '4'}, {'4', '5'}, {'4', '6'}, {'4', '7'},
      {'4', '8'}, {'4', '9'}, {'5', '0'}, {'5', '1'}, {'5', '2'}, {'5', '3'},
      {'5', '4'}, {'5', '5'}, {'5', '6'}, {'5', '7'}, {'5', '8'}, {'5', '9'},
      {'6', '0'}, {'6', '1'}, {'6', '2'}, {'6', '3'}, {'6', '4'}, {'6', '5'},
      {'6', '6'}, {'6', '7'}, {'6', '8'}, {'6', '9'}, {'7', '0'}, {'7', '1'},
      {'7', '2'}, {'7', '3'}, {'7', '4'}, {'7', '5'}, {'7', '6'}, {'7', '7'},
      {'7', '8'}, {'7', '9'}, {'8', '0'}, {'8', '1'}, {'8', '2'}, {'8', '3'},
      {'8', '4'}, {'8', '5'}, {'8', '6'}, {'8', '7'}, {'8', '8'}, {'8', '9'},
      {'9', '0'}, {'9', '1'}, {'9', '2'}, {'9', '3'}, {'9', '4'}, {'9', '5'},
      {'9', '6'}, {'9', '7'}, {'9', '8'}, {'9', '9'}};
  FMT_API static constexpr const char hex_digits[] = "0123456789abcdef";
  FMT_API static constexpr const char signs[4] = {0, '-', '+', ' '};
  FMT_API static constexpr const char left_padding_shifts[5] = {31, 31, 0, 1,
                                                                0};
  FMT_API static constexpr const char right_padding_shifts[5] = {0, 31, 0, 1,
                                                                 0};
  FMT_API static constexpr const unsigned prefixes[4] = {0, 0, 0x1000000u | '+',
                                                         0x1000000u | ' '};
};

#ifdef FMT_SHARED
// Required for -flto, -fivisibility=hidden and -shared to work
extern template struct basic_data<void>;
#endif

#if __cplusplus < 201703L
// DEPRECATED! These are here only for ABI compatiblity.
template <typename T> constexpr const char basic_data<T>::digits[][2];
template <typename T> constexpr const char basic_data<T>::hex_digits[];
template <typename T> constexpr const char basic_data<T>::signs[];
template <typename T> constexpr const char basic_data<T>::left_padding_shifts[];
template <typename T>
constexpr const char basic_data<T>::right_padding_shifts[];
template <typename T> constexpr const unsigned basic_data<T>::prefixes[];
#endif

template <typename T>
int format_float(char* buf, std::size_t size, const char* format, int precision,
                 T value) {
#ifdef FMT_FUZZ
  if (precision > 100000)
    throw std::runtime_error(
        "fuzz mode - avoid large allocation inside snprintf");
#endif
  // Suppress the warning about nonliteral format string.
  int (*snprintf_ptr)(char*, size_t, const char*, ...) = FMT_SNPRINTF;
  return precision < 0 ? snprintf_ptr(buf, size, format, value)
                       : snprintf_ptr(buf, size, format, precision, value);
}

template FMT_API dragonbox::decimal_fp<float> dragonbox::to_decimal(float x)
    FMT_NOEXCEPT;
template FMT_API dragonbox::decimal_fp<double> dragonbox::to_decimal(double x)
    FMT_NOEXCEPT;
}  // namespace detail

// Workaround a bug in MSVC2013 that prevents instantiation of format_float.
int (*instantiate_format_float)(double, int, detail::float_specs,
                                detail::buffer<char>&) = detail::format_float;

#ifndef FMT_STATIC_THOUSANDS_SEPARATOR
template FMT_API detail::locale_ref::locale_ref(const std::locale& loc);
template FMT_API std::locale detail::locale_ref::get<std::locale>() const;
#endif

// Explicit instantiations for char.

template FMT_API auto detail::thousands_sep_impl(locale_ref)
    -> thousands_sep_result<char>;
template FMT_API char detail::decimal_point_impl(locale_ref);

template FMT_API void detail::buffer<char>::append(const char*, const char*);

// DEPRECATED!
// There is no correspondent extern template in format.h because of
// incompatibility between clang and gcc (#2377).
template FMT_API void detail::vformat_to(
    detail::buffer<char>&, string_view,
    basic_format_args<FMT_BUFFER_CONTEXT(char)>, detail::locale_ref);

template FMT_API int detail::snprintf_float(double, int, detail::float_specs,
                                            detail::buffer<char>&);
template FMT_API int detail::snprintf_float(long double, int,
                                            detail::float_specs,
                                            detail::buffer<char>&);
template FMT_API int detail::format_float(double, int, detail::float_specs,
                                          detail::buffer<char>&);
template FMT_API int detail::format_float(long double, int, detail::float_specs,
                                          detail::buffer<char>&);

// Explicit instantiations for wchar_t.

template FMT_API auto detail::thousands_sep_impl(locale_ref)
    -> thousands_sep_result<wchar_t>;
template FMT_API wchar_t detail::decimal_point_impl(locale_ref);

template FMT_API void detail::buffer<wchar_t>::append(const wchar_t*,
                                                      const wchar_t*);

template struct detail::basic_data<void>;

FMT_END_NAMESPACE
```

### Quick search

### Table of Contents

- rang - Colors for your Terminal
- Zstr
- C++ API

«
hide menu

menu
sidebar
»

### Navigation

- index
- toc
- Utils »
- Program Listing for File format.cc

© Copyright 2021, Enrico Bertolazzi.
Created using Sphinx 4.2.0.
